# Therapeutic drug monitoring of anti-TNF drugs: an overview of applicability in daily clinical practice in the era of treatment with biologics in juvenile idiopathic arthritis (JIA)

**DOI:** 10.1186/s12969-021-00545-x

**Published:** 2021-04-29

**Authors:** A. Nassar-Sheikh Rashid, D. Schonenberg-Meinema, S. C. Bergkamp, S. Bakhlakh, A. de Vries, T. Rispens, T. W. Kuijpers, G. Wolbink, J. M. van den Berg

**Affiliations:** 1 Zaans Medical Center, Zaandam and Emma Children’s Hospital, Amsterdam UMC, Pediatric Immunology, Rheumatology and Infectious Diseases, University of Amsterdam, Amsterdam, The Netherlands; 2 Emma Children’s Hospital, Amsterdam UMC, Pediatric Immunology, Rheumatology and Infectious Diseases, University of Amsterdam, Amsterdam, The Netherlands; 3 Department of Immunopathology, Sanquin Diagnostic Services Amsterdam, Amsterdam, The Netherlands; 4 Department of Immunopathology, Sanquin Research and Landsteiner Laboratory, Amsterdam, The Netherlands; 5 Department of Immunopathology, CLB Sanquin Amsterdam and Department of Rheumatology, Jan van Breemen Institute Amsterdam, Amsterdam, The Netherlands

**Keywords:** Biologics, Anti-TNF drug trough levels, Anti-drug antibodies, Children, Juvenile idiopathic arthritis (JIA)

## Abstract

**Background:**

Anti-tumor necrosis factor (TNF) drugs have improved the prognosis for juvenile idiopathic arthritis (JIA) significantly. However, evidence for individual treatment decisions based on serum anti-TNF drug levels and the presence of anti-drug antibodies (ADAbs) in children is scarce. We aimed to assess if anti-TNF drug levels and/or ADAbs influenced physician’s treatment decisions in children with JIA.

**Methods:**

Patients’ records in our center were retrospectively screened for measurements of anti-TNF drug levels and ADAbs in children with JIA using etanercept, adalimumab or infliximab. Clinical characteristics and disease activity were retrieved from patient charts.

**Results:**

We analyzed 142 measurements of anti-TNF drug levels in 65 children with JIA. Of these, ninety-seven (68.3%) were trough concentrations. *N* = 14/97 (14.4%) of these showed trough concentrations within the therapeutic drug range known for adults with RA and IBD. ADAbs against adalimumab were detected in seven patients and against infliximab in one patient. Seven (87,5%) of these ADAb-positive patients had non-detectable drug levels. A flowchart was made on decisions including rational dose escalation, stopping treatment in the presence of ADAbs and undetectable drug levels, showing that 45% of measurements influenced treatment decisions, which concerned 65% of patients (*n* = 42/65).

**Conclusions:**

In the majority of patients, measurement of anti-TNF drug levels led to changes in treatment. A wide variation of anti-TNF drug levels was found possibly due to differences in drug clearance in different age groups. There is need for determination of therapeutic drug ranges and pharmacokinetic curves for anti-TNF and other biologics in children with JIA.

## Background

Juvenile idiopathic arthritis (JIA) is a heterogeneous group of diseases characterized by idiopathic chronic joint inflammation that persists for longer than 6 weeks, and has a disease onset before the age of 16 years. It is the most prevalent rheumatic disease in childhood [1]. Long term complications include chronic pain and joint damage causing physical disability and a decreased quality of life [2, 3]. In a substantial proportion of children with JIA, intermittently active disease persists into adulthood [4]. Since the introduction of biologicals, such as anti-tumor necrosis factor (TNF) drugs, treatment options have increased in children with JIA and its prognosis has improved significantly [1, 4]. Various anti-TNF drugs have been studied in children with JIA and are currently the mainstay of therapy in methotrexate-refractory JIA [5]. Biologics that are currently being used in JIA include anti-TNF drugs (etanercept, adalimumab, infliximab, golimumab and certolizumab pegol). This has changed the pediatric rheumatology landscape substantially, but improvements in the use of anti-TNF drugs are still needed, especially since anti-TNF drugs will be used earlier in the disease course when following recent international adaptations of JIA treat-to-target strategies [6].

A relationship between drug concentrations and clinical outcome has been established in adults with RA, but anti-TNF drug concentrations varied widely between subjects [7–9]. One factor contributing to the variation in drug levels is the development of anti-drug antibodies (ADAbs), a response called “immunogenicity” [7, 8]. The presence of ADAbs can lead to a diminished half-life of the drug and thus decrease its efficacy. It may also lead to adverse effects, such as hypersensitivity reactions and anaphylaxis [10]. The clinical relevance of ADAbs has again been underlined in a recent systematic review by Swart et al. [11]. Several studies have shown that the risk for development of ADAbs is inversely related to the anti-TNF drug dose and these patients often have a subtherapeutic serum trough level [12, 13]. Increasing the dosage leads to higher drug concentrations and has been shown to subsequently lead to a decrease of ADAbs [14, 15]. Higher concentrations of adalimumab and infliximab in serum have been reported to correlate with clinical response not only in adults with RA, but also in adults with psoriasis [16] or IBD [17]. Several studies have been published on children with IBD receiving anti-TNF drug treatment showing a relationship between trough levels and disease activity [18, 19]. The therapeutic range is a pharmacologic term for the concentration range that provides efficacy without resulting in unacceptable toxicity [20, 21]. The therapeutic drug ranges for anti-TNF drugs in pediatric rheumatology are not yet defined [22]. In adults with RA and ankylosing spondylitis an etanercept concentration between 2 and 3 μg/mL is seen as therapeutic [23, 24]. To reach adequate clinical response in RA, adalimumab trough levels should be in a range of 5–8 μg/mL [7]. Studies in adults with inflammatory bowel disease (IBD) have described a therapeutic drug range of 3–7 μg/mL for infliximab [25]. In a recently published observational study of Naviglio et al. in children with IBD, infliximab concentrations > 3.11 μg/mL predicted sustained clinical remission and most cases of therapeutic failure were associated with low serum drug levels [19].

Personalized medicine, which is a model for tailoring the therapeutic strategy to the needs of the individual patient, is a promising approach in health care [26]. It maximizes efficacy and minimizes drug toxicity, and seems a very promising method to use in biologic treatment regimens as well [27]. Switching between biologics, increasing dosage when there is loss of response, or tapering the dosage after achieving inactive disease on medication are all examples of decisions pediatric rheumatologists now make based on clinical experience alone. Measuring serum trough levels of biologics and monitoring the presence of blocking ADAbs could be of use in practicing personalized medicine. Currently, there is a lack of data in the literature supporting this strategy in children with JIA. We hereby present an overview of children with JIA being treated with anti-TNF drugs in our tertiary center in whom serum (mostly trough) levels and -if applicable- ADAbs were measured. We aimed to assess if therapeutic drug monitoring (TDM) was of influence in treatment decisions in children with JIA.

## Methods

We conducted a retrospective study in children under the age of 18 years with a diagnosis of JIA (excluding systemic) fitting the ILAR criteria [28] that were treated with etanercept (Enbrel®), adalimumab (Humira®) and/or infliximab (Remicade®, Remsima®, Inflectra®) in our center. As golimumab (Simponi®) had just recently been approved for use in polyarticular JIA in the Netherlands, we did not have enough data on this drug to include in our overview. The patient data were retrieved from electronic patient records of the pediatric rheumatology department of the Emma Children’s Hospital (Amsterdam University Medicals Centers). All children that had been tested for anti-TNF drug levels in the time period of 2010 up to 2020 were included in this survey. Anti-TNF drug levels were determined using validated enzyme-linked immuno sorbent assays (ELISA) as described before [7, 23, 29]. In the etanercept and adalimumab groups, (trough) drug levels were measured at a regular visit at the outpatient clinic. The definition for ‘trough’ level is the lowest concentration reached by a drug before the next dose is administered. Trough levels were defined as a drug level 1–2 days before the next administration of injection for etancercept and adalimumab. Infliximab concentrations were all trough levels as all blood samples had been taken just minutes prior to the next infusion. Trough levels were separately analyzed as subgroup per frequency regime. Cut-off levels were based on adult data, as pediatric data on therapeutic drug levels for these drugs are not yet known. In this manuscript we defined the therapeutic drug range for etanercept based on available literature in adults with RA and ankylosing spondylitis (2–3 μg/mL) [23, 24], the therapeutic drug range for adalimumab in adults with RA (5–8 μg/mL) [7] and the therapeutic drug range for infliximab on adults with RA and inflammatory bowel disease (IBD) (3–7 μg/mL) [25, 30]. ADAbs [31] were determined in all patients with low drug concentrations of adalimumab and infliximab, because in the assays we used, free (neutralizing) ADAbs will not be detected if an excess of drug is present in the serum [32]. Sometimes ADAbs were determined routinely in patients at the physician’s discretion. ADAbs were not determined in patients on etanercept, as immunogenicity does not seem to be an issue in this drug. Several studies have shown no clinical significant antibody formation to etanercept [32, 33].

The following clinical variables were collected: age, sex, JIA subtype, current medication, reason for testing and decision effect of trough level and presence of ADAbs on therapy. Secondary loss of response was defined as a relapse of arthritis in a patient with JIA after an initial response to the drug, after excluding other causes for the relapse. Partial response was defined improvement of number of active joints and/or global assessment (PGA) or parent/patient Visual Analogue Scale (VAS) but no remission. No response was defined as no improvement or worsening of arthritis.

Data entry and management was carried out anonymously, hence informed consent was waved by the medical ethical committee. Microsoft Office Excel, 2007 (Microsoft Corp, Redmond, WA, USA®) was used for data collection. Statistical analyses were carried out using IBM SPSS software (version 26).

## Results

**Fig. 1:**
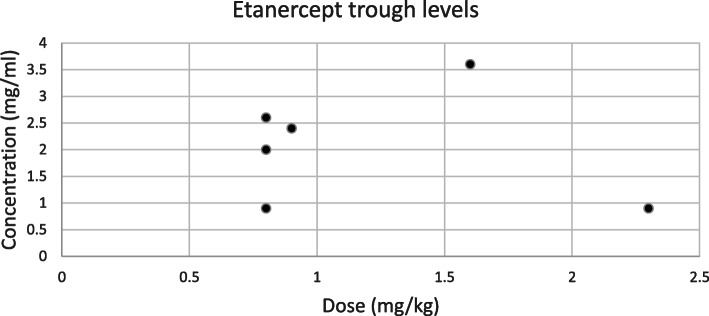
Dose vs. etanercept concentration

**Fig. 2:**
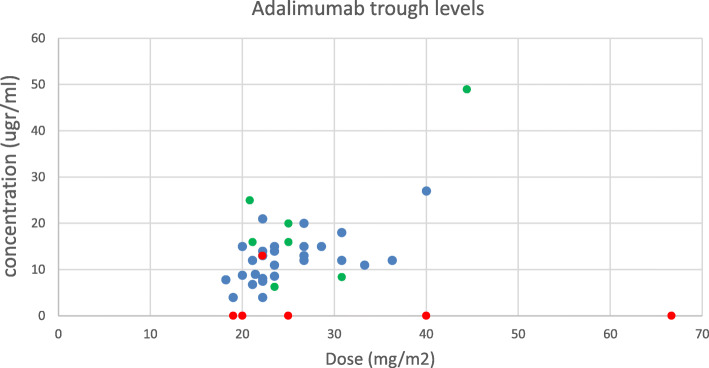
Dose vs. adalimumab concentration. Red: ADA-positive patients. Green: frequency every 7 days. Blue: frequency every 14 days

**Fig. 3:**
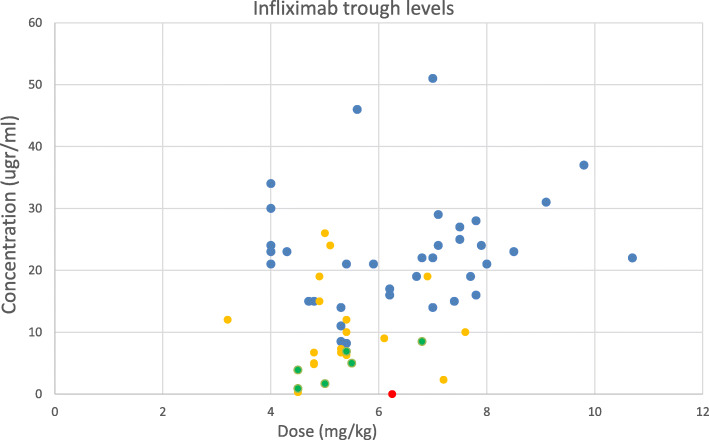
Dose vs. infliximab concentration. Red: ADA-positive patients. Blue: frequency every 4 weeks. Yellow: frequency every 6 weeks. Green: every 8 weeks

A total of 142 serum samples from 65 patients with JIA were analyzed (characteristics shown in Table 1). The number of sera samples tested per individual patient ranged from 1 to 8 (median 2, IQR: 1–3) (Table 2). The different drug levels versus drug dose (per kg or m2) are plotted in Figs. 1, 2 and 3.

**Table 1:** Patient characteristics (*N* = 65)

Age, median (range)	12.6 (2.4–17.8)
IQR	9.7–15.9
Sex (number and proportion)	
Male	19 (29%)
Female	46 (71%)
Diagnosis	
Oligo articular JIA, persistent	7 (11%)
Oligo articular JIA, extended	6 (9%)
Polyarticular JIA, RF negative	29 (45%)
Polyarticular JIA, RF positive	9 (14%)
Enthesitis-related JIA	10 (15%)
Psoriatic arthritis	3 (5%)
Undifferentiated	1 (2%)
Non-biologic DMARD use^a^	
Methotrexate	57 (78%)
Leflunomide	2 (3%)
Azathioprine	4 (6%)
Cellcept	1 (1%)
None	10 (14%)

^a^ some patients switched their non-biologic DMARD during treatment

**Table 2:** Patient drug levels and anti-drug antibodies

		Subgroup analysis with trough levels
Anti TNF, no. of drug levels/no. of patients^a^		
Etanercept	21/15	6/6
Adalimumab	51/38	22/21
Infliximab	70/28	70/28
Drug levels, median (range / IQR)		
Etanercept	2.4 (0.0–8.1 / 1.8–3)	2.2 (0.9–2.7 / 0.9–2.9)
Adalimumab	12 (0–49 / 4.8–15)	10.2 (0–27 / 0.01–14)
Infliximab	16 (0–81 / 7.3–23)	16 (0–81 / 7.3–23)
Therapeutic drug levels^b^ (% per drug group)		
Etanercept		3 (50%)
Adalimumab		0 (0%)
Infliximab		11 (16%)
Subtherapeutic drug levels^b^		
Etanercept		2 (33%)
Adalimumab		9 (41%)
Infliximab		5 (7%)
Supratherapeutic drug levels^b^		
Etanercept		1 (17%)
Adalimumab		13 (59%)
Infliximab		53 (77%)
Antidrug antibodies (% of patients using that drug)		
Adalimumab	7 (18.4%)	
Infliximab	1 (4%)	

^a^ A few patients had received more than one anti-TNF drug on different time points

^b^ according to adult data in RA and IBD; therapeutic drug levels for JIA are not yet determined

Looking at trough levels, 14.4% (*n* = 14/97) were trough levels in the therapeutic drug ranges that are known for adults in RA and IBD (Table 2). Concentrations were measured for different reasons. These included primary or secondary loss of response (55%), remission (possibility to stop or taper treatment (15.5%), uveitis flare (12%), allergic reaction (1.4%), measurement after dosage change (6.3%) or unknown reason (not explained in patient file) (9.8%).

**Fig. 4:**
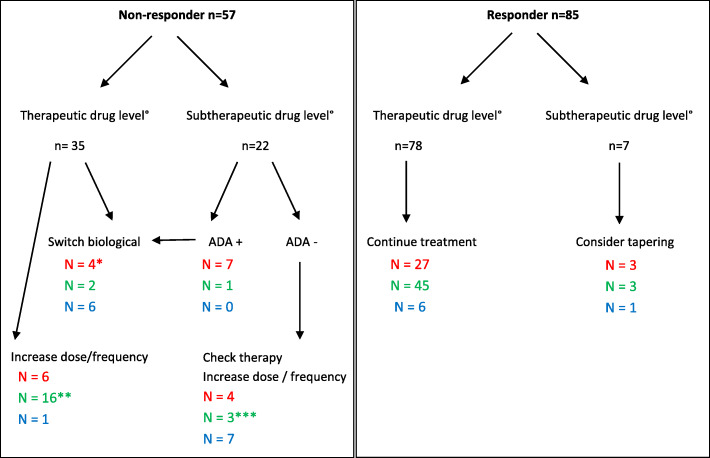
Flow chart

Trough levels with influence of treatment decisions occurred in 45% (*n* = 64/142) of measurements which concerned 65% (*n* = 42/65) of patients. These treatment changes included dose/frequency increase, or stopping and switching treatment in the presence of ADAbs combined with undetectable drug levels. We have elaborated this process in a flowchart (Fig. 4).

ADAbs were detected in 18.4% (*n* = 7/38) of patients in the adalimumab group and in one patient (4%) in the infliximab group. All but one patient with ADAbs showed non-detectable drug trough levels. One ADA+ positive patient had antibodies against adalimumab and a subtherapeutic level. After changing to higher frequency of administration (weekly instead of every 14 days), the ADA against adalimumab could not be detected anymore. Treatment failure occurred in 87.5% (*n* = 7/8) of ADA-positive measurements. One ADA-positive patient developed an anaphylactic reaction during the second infusion of infliximab. ADA and drug level was tested 1–2 h after of this complicated infusion: ADA were 310 AE/ml and infliximab drug level was non-detectable. Four months later, this same rheumatoid-factor positive JIA-patient, developed ADA against adalimumab.

## Discussion

This study shows that TDM has a role in standard follow-up of anti-TNF treatment of JIA. In the majority of patients in this study, TDM had influence on treatment decisions, even considering the fact that the therapeutic drug ranges for anti-TNF drugs in JIA are currently not established. Interpreting our data according to therapeutic drug ranges for adults with RA and IBD, only 15% would have drug levels in a therapeutic range. This implicates that there might be room for improvement in dosage regimes. One could argue that some children are undertreated, but a larger group is possibly over-treated based on the numbers of high serum levels (77% in infliximab). Another hypothesis is that therapeutic drug ranges differ from adult ranges and might be higher.

There seem to be opportunities to adapt anti-TNF drug dose to individual needs, also depending on drug clearance and treatment responses. This could potentially save costs if remission would be achieved sooner. For example, in non-responders, clinical remission could hypothetically be achieved sooner by using higher dosages or frequencies (guided by drug trough levels). This could lead to a decrease in unnecessary switching in biologics, which is relevant for children with a chronic disease in relation to future therapeutic choices and options. Switching therapy should be reserved for non-responders with high drug concentration or non-responders with low drug concentration combined with ADAbs. In case of remission (responders) and high drug levels, dosage or frequency could be tapered, which is equally important considering the high financial burden of anti-TNF drugs, but also diminishes the risk of developing side-effects. In responders with low drug concentration, stopping treatment should be considered.

A wide variation in anti-TNF drug concentrations was found in our cohort, also in subgroups with comparable dosage per kg or BSA and comparable frequencies. We did not find any differences between the JIA subtypes. In the adalimumab and infliximab group, children with JIA-associated uveitis should be viewed differently, as it has been suggested that a higher dosage of anti-TNF drug is needed for penetration to obtain adequate drug levels in the eye [34]. In our study, 21% (*n* = 6/28) of studied infliximab patients had JIA-associated uveitis, of which three patients with active uveitis. Anti-TNF drug concentrations in adults can also vary widely between subjects, even with fixed dosages of anti-TNF drugs [7–9]. It is possible that this is due to different ages of patients and its influence on drug clearance [35]. Absorption, distribution, metabolism and excretion of the drug are factors influencing drug concentrations and differ in children compared to adults [35]. Adalimumab and infliximab are monoclonal antibodies (MAbs) structurally similar to endogenous IgG and share similar pharmacokinetic properties [36]. Due to their large size and poor membrane permeability, the distribution of these drugs is confined largely to the plasma and extracellular fluid [37]. The extracellular fluid volume fraction falls rapidly after birth, while plasma volumes rise gradually, leading to a higher total body volume available for distribution in small children than in adults. As compared to adults, it has been observed that children have faster weight-normalized plasma clearance of MAbs [36]. Lower concentrations of neonatal Fc receptor (FcRn) in vascular endothelial cells, which are responsible for recycling MAbs, lead to shorter half-lives of MAbs in small children. This implies that most drugs should be dosed relatively higher in children to achieve the same drug levels as in adults. Also, it has been postulated that levels of TNF in an individual patient may vary in time. That is: a high TNF level during active disease and a low level in inactive disease. This may in turn affect the level of anti-TNF drugs, resulting in higher serum concentration of anti-TNF drugs in inactive disease as compared to active disease in the same individual, without a change in the dose [38].

Anti-TNF drugs have some degree of immunogenicity, even fully humanized ones. Etanercept seems to be an exception, as no clinical significant antibody formation to etanercept was seen in several studies [32]. High anti-infliximab antibody levels or low residual infliximab concentrations are strongly associated with acquired therapeutic resistance to infliximab in adult patients with rheumatoid arthritis [10]. Development of ADAbs can lead to a diminished half-life of all anti-TNF drugs. All but one patient in our cohort with ADAbs showed non-detectable serum trough levels, indicative of effect on *pharmacokinetics* of the drug. In these ADA-positive patients, the drug had clearly lost its efficacy, also indicative of a relevant *pharmacodynamical* effect. Treatment failure occurred in 87.5% (*n* = 7/8) of our ADA-positive patients. One ADA-positive patient developed a anaphylactic reaction during the second infusion of infliximab. Therefore, treatment failure could not (yet) be established but the drug needed to be switched due to severe allergy. When ADAbs are measured in high titers with a non-detectable drug trough level, this specific biologic has lost its therapeutic effect in this patient. The monitoring of serum trough levels and formation of ADAbs can therefore explain a (secondary) loss of therapeutic response to anti-TNF drugs. These measurements help with decisions for future treatments, either by increasing the dose or switching to another biologic when presence of ADAbs and non-detectable trough levels are found [10]. Higher starting doses of infliximab have been shown to correlate with lower incidence of ADAbs development and improved effectiveness of the drug in JIA and RA [14, 15]. This approach may also help in preventing the development of ADAbs. Another described preventive action against development of ADAbs is the concomitant use of non-biologic DMARDs, such as methotrexate or azathioprine [11, 15, 39, 40]. In our ADA-positive patients, this protective effect of non-biologic DMARD’s was not seen. ADA-positive patients concerned different JIA-subtypes, no subtype was outstanding (*n* = 1 artritis psoriatica, *n* = 1 oligo-extended JIA, *n* = 3 RF+ polyarticular JIA, *n* = 3 RF- polyarticular JIA). One of our patients had a serum through level of 0 without the development of ADAbs, but this patient was tapering the dose of etanercept. Therapeutic drug measurements could also be used in case of suspected non-compliance.

Limitations of our study are sample size (especially for etanercept) and lack of JADAS disease activity scores. In adults with rheumatoid arthritis, psoriasis and IBD, correlations between concentration of TNF inhibitors and clinical response have been reported [9, 16, 17]. As this was not the aim of this retrospective study, we could not assess if there was a relationship between therapeutic drug level and clinical outcome in children. In current practice, JIA patients that are treated with biologics are monitored by the Juvenile Arthritis Disease Activity Score (JADAS) which represents physical examination (number of active joints), physician’s VAS (Visual Analog Scale), patient’s VAS of well-being, and ESR. These data were not available for all patients at important time-points and should be included in future prospective studies for assessment of therapeutic drug range(s) of biologics. Nevertheless, with our available data we think that the importance and relevance of TDM in treatment decisions for anti-TNF drugs in JIA can be shown.

## Conclusion

TDM of anti-TNF drugs is a valuable tool in making treatment decisions in JIA such as rational dose escalation and stopping treatment in the presence of ADAbs and undetectable drug levels. This paper shows that TDM in anti-TNF is useful in daily clinical practice but we need to determine the therapeutic drug ranges and pharmacokinetic curves of anti-TNF drugs in JIA.

### Future perspective

In the era of biologics and personalized medicine there is a need for more studies on TDM in JIA treatment with biologics.

Next steps in this research area are describing anti-TNF drug pharmacokinetics (PK) specifically for children and the concentration–effect relationship of anti-TNF drug using pharmacokinetic–pharmacodynamic (PK–PD) modelling in patients with JIA.

## Data Availability

The datasets used and/or analyzed during the current study are available from the corresponding author on reasonable request.

## Abbreviations

ADAbs: Anti-drug antibodies
DMARDs: Disease Modifying Anti-Rheumatic Drugs
ELISA: Enzyme-linked immuno sorbent assay
FcRn: Fc receptor
FDA: Food and Drug Administration
IBD: Inflammatory bowel disease
IL-1: Interleukin-1
IQR: Interquartile ranges
JADAS: Juvenile Arthritis Disease Activity Score
JIA: Juvenile Idiopathic Arthritis
MAbs: Monoclonal antibodies
NSAID: Non-Steroidal Anti-Inflammatory Drug
PK: Pharmacokinetics
PK-PD: Pharmacokinetic–pharmacodynamic
RA: Rheumatoid arthritis
TDM: Therapeutic drug monitoring
TNF: Tumor necrosis factor
VAS: Visual Analog Scale
